# Long-Term Risk of Breast Cancer after Diagnosis of Benign Breast Disease by Screening Mammography

**DOI:** 10.3390/ijerph19052625

**Published:** 2022-02-24

**Authors:** Marta Román, Javier Louro, Margarita Posso, Carmen Vidal, Xavier Bargalló, Ivonne Vázquez, María Jesús Quintana, Rodrigo Alcántara, Francina Saladié, Javier del Riego, Lupe Peñalva, Maria Sala, Xavier Castells, on behalf of the BELE and IRIS Study Groups

**Affiliations:** 1 Epidemiology and Evaluation Department, IMIM (Hospital del Mar Medical Research Institute), 08003 Barcelona, Spain; jlouro@imim.es (J.L.); mposso@parcdesalutmar.cat (M.P.); msalaserra@parcdesalutmar.cat (M.S.); 2 Research Network on Health Services in Chronic Diseases (REDISSEC), 08003 Barcelona, Spain; 3 Cancer Prevention and Monitoring Program, Catalan Institute of Oncology (ICO), 08908 Barcelona, Spain; cvidal@iconcologia.net; 4 Department of Radiology, Hospital Clinic, 08036 Barcelona, Spain; xbarga@clinic.cat; 5 Pathology Department, IMIM (Hospital del Mar Medical Research Institute), 08003 Barcelona, Spain; ivazquez@psmar.cat; 6 Department of Clinical Epidemiology and Public Health, University Hospital de la Santa Creu i Sant Pau, 08025 Barcelona, Spain; mjquintana@santpau.cat; 7 Radiology Department, IMIM (Hospital del Mar Medical Research Institute), 08003 Barcelona, Spain; ralcantara@parcdesalutmar.cat; 8 Epidemiology and Cancer Prevention Service, Hospital Universitari Sant Joan de Reus, IISPV, 43204 Reus, Spain; francina.saladie@salutsantjoan.cat; 9 Department of Radiology, Parc Taulí University Hospital-UAB, 08208 Sabadell, Spain; jdelriego@tauli.cat; 10 Breast Cancer Screening Technical Office, Private Foundation Asil Hospital, 08402 Granollers, Spain; lpenalva@fhag.es

**Keywords:** breast neoplasms, mass screening, longitudinal studies, benign breast disease

## Abstract

Assessing the long-term risk of breast cancer after diagnosis of benign breast disease by mammography is of utmost importance to design personalised screening strategies. We analysed individual-level data from 778,306 women aged 50–69 years with at least one mammographic screening participation in any of ten breast cancer screening centers in Spain from 1996 to 2015, and followed-up until 2017. We used Poisson regression to compare the rates of incident breast cancer among women with and without benign breast disease. During a median follow-up of 7.6 years, 11,708 (1.5%) women had an incident of breast cancer and 17,827 (2.3%) had a benign breast disease. The risk of breast cancer was 1.77 times higher among women with benign breast disease than among those without (95% CI: 1.61 to 1.95). The relative risk increased to 1.99 among women followed for less than four years, and remained elevated for two decades, with relative risk 1.96 (95% CI: 1.32 to 2.92) for those followed from 12 to 20 years. Benign breast disease is a long-term risk factor for breast cancer. Women with benign breast disease could benefit from closer surveillance and personalized screening strategies.

## 1. Introduction

Benign breast disease is a major risk factor that doubles the risk of subsequent breast cancer [1,2]. Most women with a benign breast disease diagnosis are referred to routine breast cancer screening. At present, it remains uncertain how the long-term risks of breast cancer after a benign breast disease detected by screening evolves over time. Although different guidelines exist, there is no consensus for frequency of surveillance imaging after benign breast disease diagnosis [3,4,5].

The recent debate on personalized screening strategies and how to improve the effectiveness of breast cancer screening require reliable identification of women at risk of developing breast cancer. The debate has created a need for information on how the incidence of breast cancer after a benign breast disease varies with the characteristics of the patient, and over time. Different individualized risk prediction models for breast cancer use previous benign breast disease as a risk factor [6,7]. The long-term risk of breast cancer after a benign breast disease has important implications to design personalized strategies [8,9].

Several cohort studies analyzing the risk of breast cancer in women with benign breast disease have been conducted in recent years [1,10,11]. However, these studies either were performed in a clinical setting, have short duration of follow-up or are comprised of women with benign breast disease only, limiting their ability to compare with the average population.

Therefore, to provide further information on the long-term consequences of benign breast disease, we undertook a population-based study characterizing the risk of breast cancer after a benign breast disease among women participating at biennial screening mammography in Spain.

## 2. Materials and Methods

### 2.1. Setting and Study Population

Mammographic screening in Spain started in 1990 and achieved national coverage in 2006. It is population-based, follows the recommendations of the European Guidelines [12,13,14], and is publicly funded. The program is organized into screening centers responsible for management of breast cancer screening in their reference areas. Women in the age range 50 to 69 years are sent letters by post every two years inviting them to attend an appointment for a two-view screening mammography (craniocaudal, and mediolateral oblique) of each breast. The program has an average participation rate of 67% of invited women, and a re-attendance rate of 91.2% [15,16].

Screening mammograms are read by certified breast radiologists who interpret at least 1500 screening mammograms per year. The BI-RADS^®^ assessment categories are used to rate the probability of cancer [16,17]. Prior mammograms are available for comparison at subsequent screens. The standard procedure is double-blind reading with consensus. In cases of disagreement, double-blind reading with arbitration is used. Women with abnormal mammographic findings are recalled for further assessment to rule out malignancy. Those with negative assessment are referred back to regular screening invitation at 2 years. Further assessments include additional imaging procedures and/or biopsy.

Data for the study were obtained from ten centers of the Breast Cancer Screening Program in Spain (Cantabria, Asturias, Tarragona, Girona, Costa de Ponent (ICO), Vallès Oriental (Granollers), Vallès Occidental Est (Parc Taulí), Hospital Clinic i Provincial de Barcelona, Hospital de la Santa Creu i Sant Pau de Barcelona, Barcelona- Àrea Metropolitana Sud). The centers prospectively register information on patient-related factors, screening mammography examinations, recall, further assessments and diagnoses performed in their defined catchment areas. The resulting dataset included information about 782,352 women aged 50–69 years screened at least once between January 1996 and December 2015, with follow-up until December 2017. To be included in the study, women needed at least one negative mammographic screening prior to the mammogram that diagnosed their cancer. Thus, we excluded 4046 women with a breast cancer diagnosed at first screen. This left 778,306 women for analyses.

### 2.2. Analyses

All breast biopsies were examined and classified by hospital pathologists in each screening Centre. All biopsies with a non-malignant diagnosis were considered as benign breast disease. In order to perform subgroup analyses, benign breast diseases were classified into non-proliferative and proliferative disease, following the criteria stablished by Dupont and Page [18]. Nonproliferative disease included fibroadenomas, cysts, microcalcifications, fibrosis, apocrine, metaplasia, atrophy, fatty tissue necrosis, inflammatory tissue, and ectasia. Proliferative disease included scar, hyperplasia, sclerosing adenosis, papilloma, adenosis, intraductal hyperplasia, lobular hyperplasia, benign Phylloides tumor, benign mesenquimal tumors, epithelial benign tumors, atypia, atypical ductal hyperplasia, and lobular intraepithelial neoplasia. If a woman had more than one benign breast disease at different examinations (*n* = 654), we included the first benign breast disease occurrence. If women had more than one histological diagnosis at biopsy or they had bilateral benign disease, we selected the benign breast disease with the highest risk of breast cancer (proliferative disease > non-proliferative disease).

All invasive cancers and ductal carcinoma in situ were included as breast cancer cases. We included screen-detected cancers and interval cancers for analyses. Interval cancers were defined as breast cancers diagnosed in the interval between a non-malignant screening result and the next screening examination. Breast cancer cases are systematically identified using the screening center databases, hospital-based cancer registries, regional Minimum Data Set, and population-based cancer registries.

For each woman in the study population we defined an index screening mammogram for start of follow-up. Follow-up for women without a benign breast disease started at the date of first mammographic screening participation. Follow-up for women with a benign breast disease started at date of diagnosis of their first benign breast disease. Since we identified all interval cancers, follow-up for all study participants ended at the date of breast cancer diagnosis, or at last screening participation plus two years of follow-up, whichever came first.

Observed rates of breast cancer were calculated for women with and without benign breast disease as the number of breast cancer cases divided by the number of women in each group. We calculated observed rates for year at index mammogram (in five-year periods: 1996–2000, 2001–2005, 2006–2010, 2011–2015), age at index mammogram (in five-year age groups: 50–54, 55–59, 60–64, 65–69), and time since index mammogram (0 to ≤4, more than 4 to ≤8, more than 8 to ≤12, and more than 12 to ≤20 years). In addition, we calculated crude rate ratios of observed breast cancer rates. We used Poisson regression to estimate the rate ratios of breast cancer in women with and without benign breast disease adjusted by year at index mammogram, age at index mammogram, and time since index mammogram. We tested for interactions between the presence of benign breast disease and the adjusting factors (year, age, and time since index mammogram).

We plotted cumulative incidence rates of breast cancer by the presence or absence of a previous benign breast disease as the number of incident breast cancer cases divided by the number of women at risk over the observed 20-year time horizon. Survival curves over the 20 years study period were stratified by age at index mammogram and year at index mammogram, to test the effect of these two potential confounding variables. Confidence intervals were calculated using exact Poisson distribution [19]. We performed a sensitivity analysis where we limited the outcome to invasive breast cancer only. Women with DCIS were censored at the time of diagnosis and not considered cases for this analysis. All tests were two-sided with a 5% significance level. Statistical analyses were conducted in R 4.0.3 (R Core Team (2021). R Foundation for Statistical Computing, Vienna, Austria).

## 3. Results

### 3.1. Study Population Characteristics

By December 2015, a total of 778,306 women had been screened at least once in any of the ten participating screening centers and were included in the study. The median follow-up was 7.6 years. Fifty-nine per cent were aged below 55 years at index mammogram, 19.3% aged 55–59, 15.6% aged 60–64, and 5.4% aged 65–69 years (Table 1). By December 2017, 250,139 (32.1%) women had been followed for four years or less, 183,829 (23.6%) for four to eight years, 191,760 (24.6%) for eight to 12 years, and 152,578 (19.6%) for more than 12 years and up to 20. A total of 17,827 (2.3%) women in the study were diagnosed with a benign breast disease, and 11,708 (1.5%) had a breast cancer diagnosis. Among those with a benign breast disease, 2.5% (*n* = 442) had a breast cancer diagnosis whereas for those without benign breast disease the proportion was 1.5% (*n* = 11,226).

### 3.2. Rates of Breast Cancer among Women with and witthout a Benign Breast Disease

The overall rates of breast cancer by year, age, and time since index mammogram stratified by the presence or absence of benign breast disease are shown in Table 2. The crude rate of breast cancer decreased with more recent years at index mammogram, as it would be expected with shorter follow-up times. Women with benign breast disease showed an increased crude rate of breast cancer compared with those without independently of year, age, and time since index mammogram. Amongst women 50–54 years at reference mammogram, the crude rate of breast cancer per 1000 women was 26.1 (95% CI: 23.0 to 29.7) and 14.2 (95% CI: 13.9 to 14.6) for those with and without benign breast disease, respectively. Whereas for women aged 65–69 years, the crude rate was 11.6 (95% CI: 7.7 to 17.5) and 5.1 (95% CI: 4.5 to 5.8), respectively. Regarding time since index mammogram, the crude rate among women who had been followed four years or less was 26.5 (95% CI: 23.1 to 30.4) and 16.9 (95% CI: 16.4 to 17.4) for those with and without benign breast disease, respectively. Whereas the crude rate for women who had been followed 12 to 20 years was 11.3 (95% CI: 7.6 to 16.7) and 5.6 (95% CI: 5.2 to 6.0), respectively.

### 3.3. Cumulative Incidence of Breast Cancer by Year and Age at Index Mammogram

**Figure 1:**
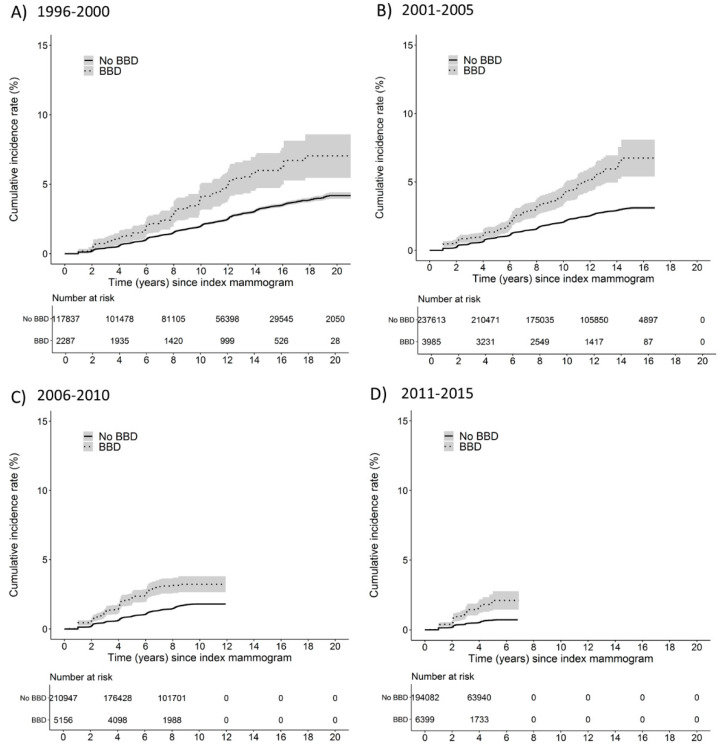
Cumulative incidence rates of breast cancer and 95% confidence intervals in women with and without benign breast disease within year at index mammogram strata; (**A**) 1996–2000, (**B**) 2001–2005, (**C**) 2006–2010, (**D**) 2011–2015. The solid line represents women without benign breast disease; the hyphen line represents women with a benign breast disease diagnosis.

Figure 1 depicts how the cumulative incidence of breast cancer cases follows a two-year step pattern produced by the biennial attendance of women in mammographic screening. The cumulative rate of breast cancer among women with benign breast disease exceeded that of those without a benign breast disease in every calendar period studied, and the observed risks continued to diverge with increasing time since the index mammogram. As a reference, by 10 years after index mammogram, the cumulative incidence rates of breast cancer for women with benign breast disease were 4.1% (95% CI: 3.2 to 5.1), 4.1% (95% CI: 3.4 to 4.8), and 3.2% (95% CI: 2.7 to 3.8), compared with 2.0% (95% CI: 2.1 to 1.9), 2.1% (95% CI: 2.1 to 2.0), and 1.8% (95% CI: 1.9 to 1.7) for women without benign breast disease during years 1996–2000, 2001–2005, and 2006–2010 at index mammogram, respectively.

**Figure 2:**
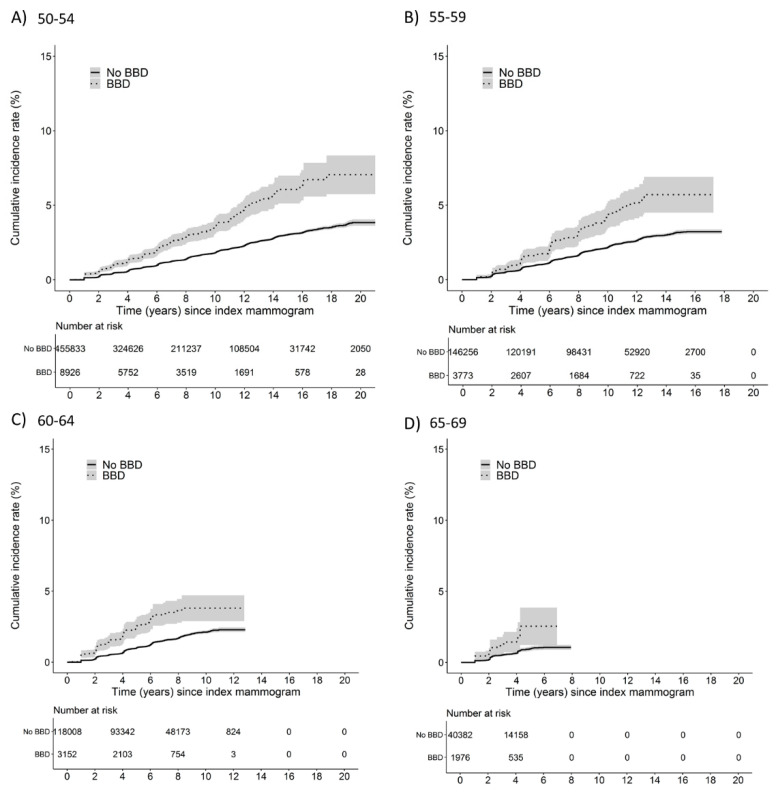
Cumulative incidence rates of breast cancer and 95% confidence intervals in women with and without benign breast disease within age at index mammogram strata; (**A**) 50–54, (**B**) 55–59, (**C**) 60–64, (**D**) 65–69. The solid line represents women without benign breast disease; the hyphen line represents women with a benign breast disease diagnosis.

Within age strata, the cumulative incidence rate of breast cancer for women with benign breast disease also diverged with increasing time (Figure 2). By 10 years after index mammogram, the cumulative incidence rates of breast cancer for women with benign breast disease were 3.6% (95% CI: 3.1 to 4.2), 4.4% (95% CI: 3.5 to 5.3) and 3.8% (95% CI: 2.9 to 4.7) compared with 1.8% (95% CI: 1.8 to 1.9), 2.2% (95% CI: 2.1 to 2.3), and 2.1% (95% CI: 2.0 to 2.2) for women without a benign breast disease at ages 50–54, 55–59, and 60–65, respectively.

### 3.4. Risk of Breast Cancer among Women with and without a Benign Breast Disease

**Figure 3:**
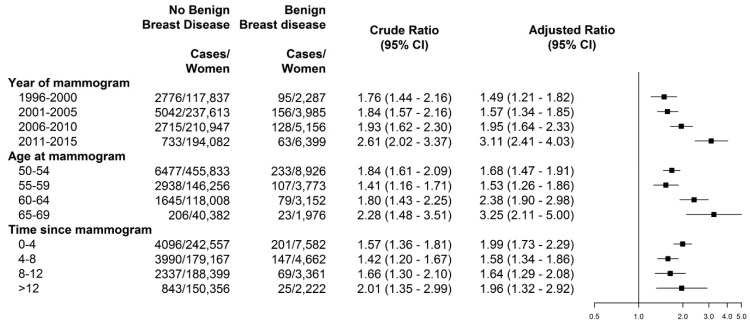
Crude and adjusted rate ratios of incidence breast cancer in women with benign breast disease at screening during January 1996 to December 2015, according to year at index mammogram, age at index mammogram, and time since index mammogram. Adjusted rate ratios by year, age, and time since index mammogram simultaneously.

Overall, women with benign breast disease had an increased risk of breast cancer (adjusted rate ratio (RR = 1.77, 95% CI: 1.61 to 1.95), compared with women without benign breast disease. The more recent the year at index mammogram, the higher the adjusted rate ratio of breast cancer for women with benign breast disease compared with women without benign breast disease, with the highest risk observed among those with index mammogram in 2011 to 2015 (RR = 3.11, 95% CI: 2.41 to 4.03) with a significant trend across calendar periods (*p*-trend < 0.05) (Figure 3). Similarly, the higher the age at index mammogram, the higher the risk in women with benign breast disease compared with women without (*p*-trend < 0.05), with the highest risk observed in women aged 65–69 years (RR = 3.25, 95% CI: 2.11 to 5.00). Interestingly, the adjusted risk comparing women with and without benign breast disease remained steady stable across time since index mammogram, with RR of 1.99 (95% CI: 1.73 to 2.29) in those with four years or less, and RR of 1.96 (95% CI: 1.32 to 2.92) in those with time 12 to 20 years, with no trend across time periods (*p*-trend = 0.97).

### 3.5. Supplementary Analyses

The analyses by benign breast disease subtype showed that women with proliferative benign breast disease had consistently increased risk compared with women with non-proliferative disease, and that the risk increased with increasing age at index mammogram for both (*p*-trend < 0.05) (Supplementary Materials, Table S1). Regarding the year at index mammogram, the highest risk was observed in more recent years for both benign breast disease subtypes.

Sensitivity analyses including invasive breast cancer only were consistent with the estimates in the full model, with modest variations in the risk estimates (Supplementary Materials, Figure S1).

## 4. Discussion

Consistent with previous studies [1,2,7,10,18,20,21,22], we have demonstrated that women with benign breast disease experienced a substantially increased risk of breast cancer. On average, women with benign breast disease had 70% higher risk of breast cancer than those without. Our results also show that the increased risk remained steady increased for 20 years after the diagnosis of benign breast disease. The increase in risk affected women of all ages in the screening age range 50–69, as well as to all periods at index mammogram. Women with proliferative benign breast disease had a higher risk than those with non-proliferative disease, and the difference in risk remained over time.

Of particular importance is the evidence that the increased risk of breast cancer persists for over 20 years after the diagnosis of benign breast disease. Since benign breast disease and its associated biopsies are frequent at breast cancer screening [16,23], benign lesions provide a means of identifying high risk women who have a long-term increased risk of breast cancer. This finding is particularly relevant to define surveillance strategies for women with benign breast disease who might be recommended for higher precision procedures like contrast-enhanced mammography or MRI.

Different from most studies [1,2,10,20,21,22], in order to correctly assess the risk of breast cancer over time, we stratified the reference population of women without benign breast disease into groups of time since index mammogram, which balances the expected to observed rates of breast cancer in the group of women with and without a benign breast disease over time. That makes a substantial difference in how the excess risk over time is compared among women with and without a benign breast disease. Using a non-stratified population of women without benign breast disease as a reference will mask the impact of benign breast disease in women with longer follow-up times, as their crude breast cancer rates are lower than those followed up for less than eight years (Table 2), but their relative risk when compared remains equally increased.

The finding that women with a previous benign breast disease had an increased risk of breast cancer that remained steady stable over 20 years suggests that benign breast lesions might be risk markers rather than precursors of subsequent breast cancer. The sustained long-term increased risk strengthens the idea of an increased proliferative nature of glandular breast tissue in some women that are more likely to develop abnormalities, which may later present as benign breast disease or malignant tumors. In a previous study we found that between 40% and 45% of breast cancer cases were contralateral to the prior benign breast lesion, which has also been shown in other studies [1,2]. Previous studies have highlighted the increased long-term risk of specific benign lesions such as fibroadenomas [22], but to our knowledge this is the first study to assess the long-term risk of breast cancer in asymptomatic screening population accounting in detail for observed follow-up time in both women with and without benign breast disease.

It should be mentioned that pathologic and diagnostic techniques have evolved during the study period. Fine needle aspiration cytology was more common at the start-up of screening programs, while today, core-needle biopsy is the preferred procedure to identify abnormalities for pathologic analyses due to improvements in techniques and accuracy. This has influenced the number of benign breast diseases diagnosed over time, but also the proportion of proliferative lesions among them, which helps to explain the increased risk associated with benign breast disease in later years (RR = 3.11 in 2011–2015 vs. RR = 1.95 in 2006–2010).

The relationship between benign breast disease and the increased risk of breast cancer is well documented in the clinical context [1,7,10,18,21]. Adding to existing evidence, we analyzed this relationship in the framework of population-based screening and therefore in asymptomatic population. In comparison with our results, a study by Tice et al. [7], also conducted in asymptomatic women, found a risk comparable to that of our study in women with non-proliferative benign breast disease. Compared with the proliferative disease category used in our study, they found a lower risk in women with proliferative disease without atypia and a higher risk in those with proliferative risk with atypia, which is expected when compared to our grouped category that includes proliferative lesions with and without atypia. On the other hand, Hartmann et al. [1] reported a somewhat lower overall risk of breast cancer in women with benign breast disease. The difference might be partially explained by the inclusion of younger women in Hartman’s study. Our study included only women in the target age rage for screening; i.e., 50 to 69 years, who are known to have a higher risk of breast cancer than younger women, and a larger proportion of menopausal women. A previous study showed that the risk of breast cancer for women with benign breast disease was greater among postmenopausal women [24]. Comparison with other studies is difficult because women with non-proliferative diseases are often used as a referent group [10,18,21].

Most individualized risk prediction models for breast cancer include previous benign breast disease as a risk factor, such as the Breast Cancer Surveillance Consortium, and Tyrer-Cuzick models [7,25,26,27]. Benign breast disease increased the risk by approximately two-fold compared to women without for predictions over 5 and 10 years. The finding in this study that the increased risk of breast cancer after a benign breast disease is sustained over time strengthens their validity for risk prediction for long periods of time.

Data for the study were collected from a consolidated population-based screening program that has been operating for more than 20 years, with an average participation of 67% of invited women, and a re-attendance rate of 91.2%, which is a major strength of this study [16]. This study characterizes the long-term risks of breast cancer in women diagnosed with benign breast disease using individual-level data from a large population-based screening program.

This study has several limitations. In our analyses, we have made efforts to control for confounding factors. However, additional information on variables such as body mass index, hormone therapy use, and menopausal status was lacking because the screening centers did not collect this information. Also, we lacked information on socioeconomic and demographic factors that are known to also be associated with the risk of breast cancer. Adjusting for these and other risk factors would have been desirable and could have refined our estimates. Nevertheless, we expect that these variables have a modest effect on the association between a benign breast disease and the risk of breast cancer, and the bias introduced by this lack of adjustment would be small. Another limitation is that information on the benign breast disease subtype was missing on 32.9% of benign breast disease diagnoses, limiting our ability to draw conclusions regarding benign breast disease subtypes. The lack of an exact histology of benign breast disease diagnosed at screening is common due to fine needle aspiration biopsies. Also, the number of incident breast cancer cases after diagnosis of a non-proliferative and proliferative benign breast disease was relatively small, again limiting our ability to identify robust associations for sub-group analyses. Despite these weaknesses, the overall quality of the data in our study is high and we were able to perform robust analyses on the impact of benign breast disease. Lastly, during the study period, there was a transition from screen-film mammography to full-field digital mammography. Full-field digital mammography was introduced in 2004 and gradually became widespread. The transition from film to digital mammography might have had an effect on recall rates, and consequently on benign breast disease and cancer detection rates. However, previous studies have shown that these outcomes were not affected by the introduction of digital mammography [28,29].

## 5. Conclusions

We have provided evidence of the long-term nature of the risk of breast cancer after diagnosis of benign breast disease by screening mammography. The increase in risk was sustained for at least 20 years after diagnosis, and affected women of all ages as well as to all years at index mammogram. Our results also show that women who had a proliferative benign disease had a higher long-term risk than those with non-proliferative disease. Women with a diagnosis of benign breast disease could benefit from closer surveillance and more personalized screening strategies.

## Figures and Tables

**Table 1:** Characteristics of women in the study population for women with and without a benign breast disease. Values are number of women (percentage).

		No BBD (*n* = 760,479)	BBD (*n* = 17,827)	Total (*n* = 778,306)
Year at index mammogram				
	1996–2000	117,837 (15.5%)	2287 (12.8%)	120,124 (15.4%)
	2001–2005	237,613 (31.2%)	3985 (22.4%)	241,598 (31.0%)
	2006–2010	210,947 (27.7%)	5156 (28.9%)	216,103 (27.8%)
	2011–2015	194,082 (25.5%)	6399 (35.9%)	200,481 (25.8%)
Age at index mammogram				
	50–54	455,833 (59.9%)	8926 (50.1%)	464,759 (59.7%)
	55–59	146,256 (19.2%)	3773 (21.2%)	150,029 (19.3%)
	60–64	118,008 (15.5%)	3152 (17.7%)	121,160 (15.6%)
	65–69	40,382 (5.3%)	1976 (11.1%)	42,358 (5.4%)
Time since index mammogram				
	≤4 years	242,557 (31.9%)	7582 (42.5%)	250,139 (32.1%)
	>4 and ≤8 years	179,167 (23.6%)	4662 (26.2%)	183,829 (23.6%)
	>8 and ≤12 years	188,399 (24.8%)	3361 (18.9%)	191,760 (24.6%)
	>12 years	150,356 (19.8%)	2222 (12.5%)	152,578 (19.6%)
Breast Cancer				
	No	749,213 (98.5%)	17,385 (97.5%)	766,598 (98.5%)
	Yes	11,266 (1.5%)	442 (2.5%)	11,708 (1.5%)

**Table 2:** Overall rates of breast cancer by the presence or absence of benign breast disease and by year, age, and time at index mammogram.

	No Benign Breast Disease			Benign Breast Disease		
	Women	Number of Breast Cancer cases	Rate per 1000 Women	Women	Number of Breast Cancer Cases	Rate per 1000 Women
Year at index mammogram						
1996–2000	117,837	2776	23.6 (22.7–24.5)	2287	95	41.5 (34.0–50.8)
2001–2005	237,613	5042	21.2 (20.6–21.8)	3985	156	39.1 (33.5–45.8)
2006–2010	210,947	2715	12.9 (12.4–13.4)	5156	128	24.8 (20.9–29.5)
2011–2015	194,082	733	3.8 (3.5–4.1)	6399	63	9.8 (7.7–12.6)
Age at index mammogram						
50–54	455,833	6477	14.2 (13.9–14.6)	8926	233	26.1 (23.0–29.7)
55–59	146,256	2938	20.1 (19.4–20.8)	3773	107	28.4 (23.5–34.3)
60–64	118,008	1645	13.9 (13.3–14.6)	3152	79	25.1 (20.1–31.2)
65–69	40,382	206	5.1 (4.5–5.8)	1976	23	11.6 (7.7–17.5)
Time since index mammogram						
≤4 years	242,557	4096	16.9 (16.4–17.4)	7582	201	26.5 (23.1–30.4)
>4 and ≤8 years	179,167	3990	22.3 (21.6–23.0)	4662	147	31.5 (26.8–37.1)
>8 and ≤12 years	188,399	2337	12.4 (11.9–12.9)	3361	69	20.5 (16.2–26.0)
>12 years	150,356	843	5.6 (5.2–6.0)	2222	25	11.3 (7.6–16.7)

## Data Availability

The datasets generated and analysed during the current study are not publicly available but are available from the authors on reasonable request.

## Supplementary Materials

The following supporting information can be downloaded at: https://www.mdpi.com/article/10.3390/ijerph19052625/s1, Figure S1: Crude and adjusted rate ratios of invasive breast cancer in women with benign breast disease at screening during January 1996 to December 2015, according to year at index mammogram, age at index mammography and time since index mammography. Adjusted rate ratios by year, age, and time since index mammogram simultaneously; Table S1: Overall rates of breast cancer in women without a benign breast disease and adjusted rate ratios of breast cancer by subtype of benign breast disease by year, age, and time at index mammogram.
