# Supplementary material for: Long-Term Risk of Breast Cancer after Diagnosis of Benign Breast Disease by Screening Mammography
Source: Int J Environ Res Public Health. 2022 Feb 24;19(5):2625. doi: 10.3390/ijerph19052625 (PMC8909630; doi:10.3390/ijerph19052625)
Supplement: Supplementary file 1 [file ijerph-19-02625-s001.zip › Supplementary material Figure 1.pdf]

	No Benign Breast Disease Cases/ Women	Benign Breast disease Cases/ Women	Crude Ratio (95% CI)	Adjusted Ratio (95% CI)
**Year of mammogram**				
1996-2000	2079/117,837	66/2,287	1.64 (1.28 - 2.09)	1.38 (1.08 - 1.77)
2001-2005	3568/237,613	110/3,985	1.84 (1.52 - 2.22)	1.58 (1.31 - 1.91)
2006-2010	1905/210,947	102/5,156	2.19 (1.79 - 2.67)	2.22 (1.82 - 2.70)
2011-2015	512/194,082	45/6,399	2.67 (1.97 - 3.62)	3.17 (2.34 - 4.30)
**Age at mammogram**				
50-54	4549/455,833	166/8,926	1.86 (1.60 - 2.18)	1.71 (1.46 - 1.99)
55-59	2125/146,256	80/3,773	1.46 (1.17 - 1.82)	1.59 (1.27 - 1.98)
60-64	1235/118,008	56/3,152	1.70 (1.30 - 2.22)	2.25 (1.72 - 2.94)
65-69	155/40,382	21/1,976	2.77 (1.76 - 4.37)	3.99 (2.53 - 6.29)
**Time since mammogram**				
0-4	2725/242,557	148/7,582	1.74 (1.47 - 2.05)	2.20 (1.86 - 2.59)
4-8	2982/179,167	99/4,662	1.28 (1.04 - 1.56)	1.42 (1.16 - 1.73)
8-12	1744/188,399	55/3,361	1.77 (1.35 - 2.31)	1.75 (1.34 - 2.29)
>12	613/150,356	21/2,222	2.32 (1.50 - 3.58)	2.26 (1.46 - 3.49)

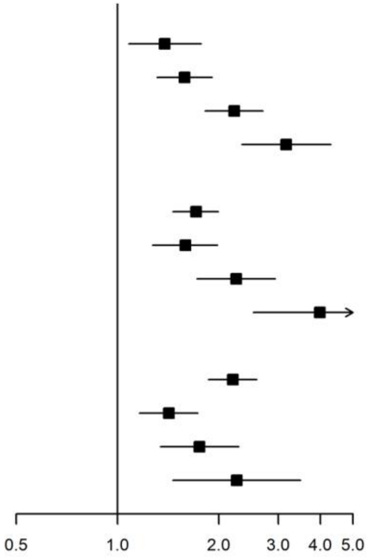

Figure S1. Crude and adjusted rate ratios of invasive breast cancer in women with benign breast disease at screening during January 1996 to December 2015, according to year at index mammo-gram, age at index mammography and time since index mammography. Adjusted rate ratios by year, age, and time since index mammogram simultaneously.
