# Supplementary material for: Long-Term Risk of Breast Cancer after Diagnosis of Benign Breast Disease by Screening Mammography
Source: Int J Environ Res Public Health. 2022 Feb 24;19(5):2625. doi: 10.3390/ijerph19052625 (PMC8909630; doi:10.3390/ijerph19052625)
Supplement: Supplementary file 1 [file ijerph-19-02625-s001.zip › Supplementary material Table 1.pdf]

**Table S1.** Overall rates of breast cancer in women without a benign breast disease and adjusted rate ratios of breast cancer by subtype of benign breast disease by year, age, and time at index mammogram.

| | No benign breast disease | Unknown | | Non-proliferative | | Proliferative | |
|---|---|---|---|---|---|---|---|
| | Cases/ Women | Cases/Women | Adjusted Rate Ratio (95%CI) | Cases/ Women | Adjusted Rate Ratio (95%CI) | Cases/Women | Adjusted Rate Ratio (95%CI) |
| Year at index mammogram | | | | | | | |
| 1996-2000 | 2 776/ 117 837 | 41/ 1 180 | 1.37 (1.01-1.87) | 41/ 945 | 1.45 (1.06-1.97) | 13/ 162 | 2.31 (1.34-3.98) |
| 2001-2005 | 5 042/ 237 613 | 50/ 1 379 | 1.55 (1.17-2.05) | 78/ 2 092 | 1.47 (1.17-1.84) | 28/ 514 | 2.03 (1.40-2.95) |
| 2006-2010 | 2 715/ 210 947 | 40/ 1 695 | 1.89 (1.38-2.58) | 62/ 2 602 | 1.87 (1.45-2.40) | 26/ 859 | 2.32 (1.58-3.41) |
| 2011-2015 | 733/ 194 082 | 14/ 1 620 | 2.74 (1.61-4.64) | 35/ 3 609 | 3.08 (2.20-4.33) | 14/ 1 170 | 3.72 (2.19-6.32) |
| Age at index mammogram | | | | | | | |
| 50-54 | 6 477/ 455 833 | 78/ 2 928 | 1.68 (1.34-2.10) | 112/ 4 628 | 1.55 (1.29-1.87) | 43/ 1 370 | 2.14 (1.59-2.89) |
| 55-59 | 2 938/ 146 256 | 36/ 1 301 | 1.36 (0.98-1.89) | 47/ 1 895 | 1.39 (1.04-1.85) | 24/ 577 | 2.50 (1.67-3.73) |
| 60-64 | 1 645/ 118 008 | 27/ 1 071 | 2.03 (1.39-2.97) | 41/ 1 607 | 2.57 (1.88-3.50) | 11/ 474 | 2.79 (1.54-5.06) |
| 65-69 | 206/ 40 382 | 4/ 574 | 1.80 (0.67-4.85) | 16/ 1118 | 3.98 (2.39-6.62) | 3/ 284 | 3.61 (1.15-11.28) |
| Time since index mammogram | | | | | | | |
| ≤ 4 years | 4 096/ 242 557 | 57/ 2 042 | 1.76 (1.35-2.28) | 109/ 4 167 | 2.09 (1.73-2.53) | 35/ 1 373 | 2.13 (1.53-2.98) |
| > 4 and ≤ 8 years | 3 990/ 179 167 | 50/ 1 533 | 1.53 (1.16-2.02) | 69/ 2 478 | 1.41 (1.11-1.79) | 28/ 651 | 2.41 (1.66-3.50) |
| > 8 and ≤ 12 years | 2 337/ 188 399 | 28/ 1 328 | 1.59 (1.10-2.31) | 28/ 1 558 | 1.46 (1.00-2.12) | 13/ 475 | 2.45 (1.42-4.23) |
| >12 years | 843/ 150 356 | 10/ 971 | 1.75 (0.94-3.26) | 10/ 1045 | 1.70 (0.91-3.17) | 5/ 206 | 4.38 (1.82-10.55) |
